# Behavioural responses of a trans-hemispheric migrant to climate oscillation

**DOI:** 10.1098/rspb.2024.1944

**Published:** 2024-10-30

**Authors:** Katrina Siddiqi-Davies, Joe Wynn, Oliver Padget, Patrick Lewin, Natasha Gillies, Joe Morford, Lewis Fisher-Reeves, Paris Jaggers, Greg Morgan, Jóhannis Danielsen, Holly Kirk, Annette Fayet, Akiko Shoji, Sarah Bond, Martyna Syposz, Lou Maurice, Robin Freeman, Ben Dean, David Boyle, Tim Guilford

**Affiliations:** ^1^ Department of Biology, University of Oxford, Mansfield Road, Oxford OX1 3SZ, UK; ^2^ Institut für Vogelforschung ‘Vogelwarte Helgoland’ An Der Vogelwarte 21, Wilhelmshaven 26386, Germany; ^3^ School of Environmental Sciences, University of Liverpool, Jane Herdman Building, Liverpool L69 3GP, UK; ^4^ Royal Society for the Protection of Birds, Pembrokeshire Coast National Park, Haverfordwest, St Davids SA62 6PY, UK; ^5^ Faroe Marine Research Institute Nóatún 1, PO Box 305, Tórshavn FO 110, Faroe Islands; ^6^ Interdisciplinary Conservation Science Group, RMIT University, La Trobe Street, Melbourne, Victoria 3000, Australia; ^7^ Norwegian Institute for Nature Research, Høgskoleringen 9, Trondheim 7034, Norway; ^8^ Nagoya University, Graduate School of Environmental Studies, Nagoya, Aichi 464-8601, Japan; ^9^ School of Ocean Sciences, Bangor University, Askew St, Menai Bridge, Bangor LL59 5AB, UK; ^10^ Department of Vertebrate Ecology and Zoology, Faculty of Biology, University of Gdańsk, Wita Stwosza 59, Gdańsk 80-308, Poland; ^11^ British Geological Survey, MacLean Building, Benson Lane, Crowmarsh Gifford, Wallingford OX10 8ED, UK; ^12^ Zoological Society of London, London NW1 4RY, UK

**Keywords:** behaviour, biologging, climate, El Niño–Southern Oscillation, migration, seabird

## Abstract

Large-scale climatic fluctuations, such as the El Niño–Southern Oscillation, can have dramatic effects on ocean ecosystem productivity. Many mobile species breeding in temperate or higher latitudes escape the extremes of seasonal climate variation through long-distance, even trans-global migration, but how they deal with, or are affected by, such longer phased climate fluctuations is less understood. To investigate how a long-lived migratory species might respond to such periodic environmental change we collected and analysed a 13 year biologging dataset for a trans-equatorial migrant, the Manx shearwater (*Puffinus puffinus*). Our primary finding was that in El Niño years, non-breeding birds were at more northerly (lower) latitudes than in La Niña years, a response attributable to individual flexibility in migratory destinations. Daily time spent foraging varied in concert with this latitudinal shift, with birds foraging less in El Niño years. Secondarily, we found that in subsequent breeding, a hemisphere away, El Niño years saw a reduction in foraging time and chick provisioning rates: effects that could not be attributed to conditions at their breeding grounds in the North Atlantic. Thus, in a highly migratory animal, individuals may adjust to fluctuating non-breeding conditions but still experience cascading carry over effects on subsequent behaviour.

## 1. Introduction

Migration can be a mechanism to escape the extremes of seasonal climate variation through long-distance, trans-hemispheric movements [1]. How these movements respond to, or are affected by, longer phased climate fluctuations that affect environmental productivity at migratory destinations is still poorly understood. One of the major drivers of climate is the El Niño–Southern Oscillation (ENSO), a climatic pattern that alternates between an El Niño state, where Pacific trade winds weaken, and the reverse, La Niña, both of which alter winds and temperatures globally [2,3]. With a periodicity of 3–7 years, both ENSO phases can influence the distribution of food resources at foraging grounds [4]. The extent to which individual animals can adapt their foraging distributions in response to shifting ENSO phases between years remains unclear, necessitating the utilization of long-term datasets of highly mobile species [5].

Long-distance migrants such as seabirds often spend non-breeding periods in productive regions, far from their high latitude or temperate breeding grounds. Breeding is energetically demanding for seabirds, which invest large amounts of parental care into rearing a small number of chicks, with breeding periods often lasting many months [6–8]. Non-breeding foraging is important for restoring condition and preparing for the following breeding season [9,10]. Both ENSO phases have been shown to affect seabirds adversely during the breeding season, reducing survival in Cory’s shearwaters (*Calonectris borealis*) during La Niña [11] causing an increase in birds skipping breeding in El Niño years for red-footed boobies (*Sula sula*) [12] and changing the at-sea breeding distribution of multiple species of tropical petrel [13]. However, it is not well understood how non-breeding ENSO conditions might impact subsequent breeding across hemispheres, where different environmental conditions are encountered. Events in one season that impact behaviour in the next are termed carry-over effects [14,15], and can occur at any stage of the annual cycle. There are many documented cases of carry-over effects in seabirds, including breeding success affecting non-breeding phenology [8,16], and increased non-breeding mass [10,17] and foraging success [18] improving breeding performance.

The Manx shearwater (*Puffinus puffinus*), a small (400 g) Procellariform seabird breeding mainly in northern Europe, is a species particularly suited to studying individual responses to ENSO owing to its high breeding site philopatry, high year-to-year survival and tolerance of bird- and nest-borne instrumentation [19]. In addition, carry-over effects are thought to be especially important for migratory species such as Manx shearwaters that undergo a long, and potentially costly trans-equatorial migration to the Patagonian shelf [20,21], and link pelagic ecology at a global scale. Manx shearwaters are long-lived with protracted breeding seasons, so must balance reproductive and survival decisions from year to year [19]. Shearwaters that spent more time foraging during the non-breeding period were found to be more likely to skip breeding that year [22]. This increased the likelihood of rearing a chick successfully in the following breeding season. In turn, breeding season conditions are known to carry-over to non-breeding behaviour and future breeding success in this species, with experimentally shortened or extended breeding seasons in Manx shearwaters having knock-on consequences that can be measured using geolocators [10].

To understand individual responses to ENSO, and whether these responses carry over into future seasons, long-term datasets of annual movements are essential [23]. To determine whether, and if so how, ENSO phase predicts non-breeding latitude, we analyse a 13-year dataset of geolocator logger-derived migratory positions and behavioural activity in the Manx shearwater. We investigate the mechanistic drivers of ENSO-related shifts by employing a mixed-effects model to assess whether birds exhibit latitude shifts in tandem with peaks in chlorophyll distribution, serving as an indicator of resource distribution. Further, we take advantage of long-term tracking of individuals, parsing out within-individual effects to investigate whether individuals are flexibly adjusting their location between years with ENSO [24]. Secondarily, we predict that effects of ENSO on shearwater non-breeding location and behaviour will carry over into the subsequent breeding season’s foraging, chick provisioning behaviour and phenology. To explore how ENSO affects shearwater migration (northbound and southbound) and breeding behaviour, we use a path analysis approach. This method allows us to simultaneously assess multiple temporally linked correlations between phenology, non-breeding location, foraging behaviour and environmental covariates [25]. Lastly, to distinguish carry-over effects from correlations between local conditions at the non-breeding and breeding sites, we construct a mixed effects model to investigate variation in breeding season foraging with the North Atlantic Oscillation (NAO), the major determinant of local conditions in the Northern Hemisphere [26]. To summarize a set of environmental predictors (e.g. sea surface temperature, precipitation and sea surface level) attributable to oscillations, we use large-scale climate indexes; the Southern Oscillation Index (SOI) (an indicator of ENSO conditions) and the NAO Index [27,28].

## 2. Methods

### (a) Fieldwork

To determine the migratory timing, routes and destinations of individual breeding birds, from 2007 to 2021, 770 global location sensor (GLS) devices were deployed and retrieved from Manx shearwaters breeding at multiple colonies across the core breeding range of the species: Rum (Scotland) (57.01°N, −6.33°E), Skomer (Wales) (51.74°N, −5.29°E), Ramsey (Wales) (51.74°N, 5.29°E), Copeland (Northern Ireland) (54.68°N, −5.53°E), Nolsoy (Faroes) (61.98°N, −6.65°E) and Lundy (England) (51.18°N, −4.67°E). To estimate daily foraging, resting and flight behaviour, we used devices that incorporated a saltwater immersion logger. Models of GLS included BAS Mk 6, 9, 15, 19 (2.5 g), BAS Mk 13, 14, 18 (1.5 g) and MigrateTech intigeo C330, C250 (3.3 g), C65, C65-Super (1 g) combined immersion and light loggers. With the average bird mass being 400 g [19], all models weighed < 1% of the birds’ total body weight. GLS devices were attached to a custom-made Darvic leg ring, using cable ties and a small amount of superglue. Handling time was typically 5 to 10 min per deployment. Although GLS devices typically can record 3 years of data, most devices were retrieved, downloaded and redeployed each year to maximize data collection. For a subset of Skomer birds, chick peak masses were obtained by daily chick weighing from 2012 to 2019 (*n* = 63 chicks).

### (b) Processing position data

All processing and statistical analyses were carried out in RStudio v. 4.0.2 [29]. Light data were processed using the ‘geolight’ package to calculate position from twilight events defined by a light intensity threshold of 10 lux [30]. Day length was used to estimate latitude, and the timing of midday/midnight was used for longitude. As light sensors may differ between geolocator models, the sun elevation angle used to define twilight events was selected independently for each individual track. Latitude versus time plots were analysed across a range of sun elevation angles to identify the one that best calculated latitudes matching the accurate breeding latitude during the summer months [31]. The selected sun elevation angle ranged from −3 to −5. A rolling 3-day mean was applied to both longitude and latitude to smooth out error [32]. Following the filtering of data to include only those that had complete tracks of north and southbound migration there were 423 bird-years available from 222 individuals. Mean January position was used to represent non-breeding foraging ground location, as it is a mid-point month where position is least likely to be affected by birds arriving from or departing on southbound and northbound migration, respectively (see electronic supplementary material 3 for more information on shearwater phenology). Given the noise associated with GLS position estimates, latitude and longitude outliers were removed using the interquartile range method, retaining the lower and upper bounds of data [33]. Migration phenology was determined using changes in longitude, rather than changes in overall position, as it is not subject to equinox error. Migration dates were determined from visual inspection of longitude (as in electronic supplementary material figure S1).

### (c) Processing immersion data

Saltwater immersion data recorded at 10 min intervals were used to measure behavioural activity. Saltwater immersion was recorded every 3 s and summarized every 10 min to form an immersion score from 0 (completely dry) to 200 (completely immersed). Geolocator models that recorded immersion at alternate bin frequencies were excluded from analysis of behaviours due to concerns over differences in observed sensitivity between devices leaving 229 complete immersion tracks, 89 of which had a consecutive year in which to assess carry over effects due to limitations of the immersion logger memory. For the times when the bird was at sea (see below for how colony visits were determined during breeding), immersion bins were classified into three states; a dry state (flying) where immersion score equalled zero, a wet state (resting/preening) where immersion score equalled its maximum and an intermediate score (foraging) that represented all values in-between. Three behavioural states have previously been identified for Manx shearwaters using immersion data in a number of different studies, with the intermediate state representing foraging [10,20,34]. Simultaneously deployed dive logger, Global Positioning System (GPS) and GLS devices validate these behaviour states, showing foraging behaviours assigned from immersion data do indeed contain most diving [35]. Foraging effort (proportion of daily time spent foraging) was obtained for non-breeding birds during January and breeding birds during August (chick rearing period). Chick rearing birds were included in the path analysis if there was evidence that breeding was successfully attempted. When direct evidence of breeding at the colony was not available, immersion data were carefully reviewed for signs of regular incubation stints, identified by characteristic extended dry periods (of at least 3 days) prior to chick rearing excluding nine non-breeding individuals. Of these, four had loggers attached during the previous non-breeding season, and they were included in a supplementary analysis exploring the relationship between skipped breeding seasons and non-breeding foraging behaviour. Manx shearwaters are known to primarily forage during daylight hours [36–38]. To standardize foraging effort for variation in day length, the number of hours at sea spent foraging were divided by day length at each bird’s mean monthly position for January foraging, and per the mean number of daylight hours at each breeding colony in August.

Colony visitation during August (a month when breeding adults across all colonies will be chick-rearing) was obtained from the immersion data to indicate chick provisioning rates. Manx shearwaters only arrive at or depart from the colony during the night, when it is dark enough to avoid predation [19,39]. If they remain present in their burrow during daylight hours, they are unlikely to depart until it is night. Therefore, during the day if there was a continuous dry period for 6 h or more, it was assumed that the bird was in its burrow. Sunrise and sunset times were derived from the R suncalc package [40]. Determining night visits to the colony required a different approach, as dark and dry periods at night could be easily confused with night flight. For each night, immersion bins were defined as ‘wet’ if any immersion was recorded and summed to calculate the number of wet events per night [41,42]. A normal expectation maximization mixture model, a model used to identify the distribution to which observations belong, was applied to distinguish nights with colony visits from nights at sea using the mixtools package [43]. Two distinct distributions were identified and hence used to identify colony visitation by assigning colony visits to nights that had a higher probability of belonging to the drier peak (see electronic supplementary material 2 for more details).

### (d) Environmental variables

Non-breeding conditions were described using the SOI, while breeding conditions were described via the NAO index, both provided by National Oceanic and Atmospheric Administration (NOAA) [28]. The SOI describes a standardized difference between the barometric pressures at observation sites in Darwin and Tahiti. When the pressure difference weakens, El Niño conditions occur, indicated by negative index values. For this analysis, the SOI index was taken as a mean for the months of October, November and December. These months coincide with the peak of an El Niño/La Niña event and phytoplankton blooms in the Southwestern Atlantic that dictate non-breeding conditions [44]. The NOA index describes the pressure difference between the Azores and Iceland. NAO is most pronounced in winter and can have effects in subsequent seasons [45]. Summer NAO has an effect on European climate but it is less understood [46]. Therefore, in this analysis, winter NAO (December–March) and summer NAO (June–August) were both considered as drivers of climate around the breeding colony. We used Aqua MODIS-derived chlorophyll a data provided by NASA to determine which latitude in the Patagonian shelf area (a box of coordinates −35°N, −63°E : −45°N, −59E°) had the maximum chlorophyll per January each year [47].

### (e) Statistical analysis

**Figure 1.**
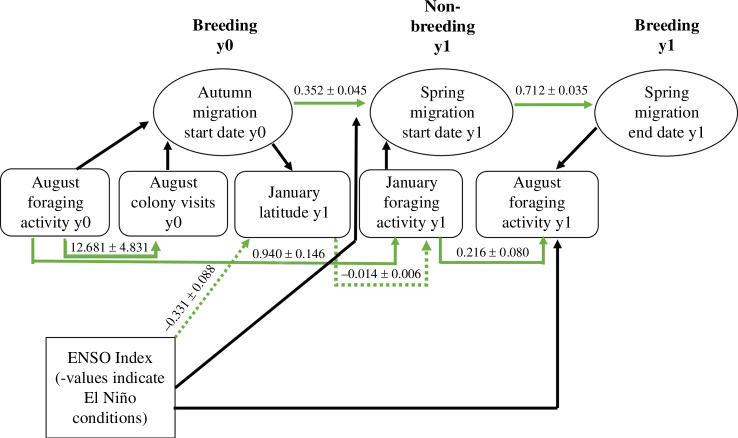
A diagram illustrating path analysis correlations between environmental, behavioural and phenological factors. All significant effects are represented in green and non-significant paths in black. Dotted lines indicate negative relationships and path estimates (ß ± s.e.) are given for each significant path.

We implemented a path analysis model to link behavioural responses from one season to the next via a path of correlated events [25,48]. This approach refines and expands on earlier work that described links across the annual behavioural cycle of this species using structural equation models, undertaken on a much smaller dataset [49]. Path analysis was conducted via the R package Lavaan to investigate links between the SOI and previous breeding season behaviour to non-breeding latitude, phenology and foraging effort [50]. These variables were then linked to the following breeding season’s behaviour via colony visitation and foraging effort during August (figure 1). Significance levels were Bonferroni adjusted for structural equation modelling [51]; $\alpha \left(per\;test\right)=0.05/k^{1-\sqrt{\left[r\right]}}\;$, where *k* is equal to the number of tests and *r* to the correlation coefficient. To avoid model overfitting, and because our analysis focuses on within individual changes between years, we did not include colony as a factor. As we were interested in determining the environmental factors causing latitudinal variation between years, and these are confounded with time, the year itself was not included in the path analysis model.

Path analysis relies on several, rather than a single statistical test to assess model fit to the data. As chi-squared (*χ*^2^) *p*-values are known to be uninterpretable with large sample sizes, we instead used the relative/normed chi-square (*χ*^2^/d.f.) to assess fit [52]. The *χ*^2^/d.f. value was 2.49, which was suitably below the recommended maximum ratio of 5. Both the comparative fit index and the Tucker–Lewis Index of the path analysis were 0.9. The root mean square error of approximation and the standardized root mean square residual, which both relate to model residuals, were 0.05 and 0.07, respectively. All of the above statistics were therefore well within the accepted thresholds for suitable model fit [53].

Separate to our path analysis, the relationship between August colony visits and chick peak mass was tested. This was to validate whether GLS-derived colony visits are indicative of chick provisioning rates in this study, following previous validation in a study using mixture models to indicate chick provisioning [41]. Chick peak mass data were available only for a subset of Skomer geolocator birds over the years of this study (*n* = 63), so all available August chick peak mass data on Skomer were pooled to increase the sample size and analysed separately using a mixed effects model in the R package lme4 with burrow as a random effect [54]. The date the peak mass was taken was included as a fixed effect to assure that any correlations between colony visitation and peak mass were not occurring as a function of peak mass being obtained later in some birds. We also implemented a binomial generalized linear model in a supplementary analysis of whether January foraging increased the likelihood of skipping breeding. Additionally, to test for local environmental conditions during breeding, the relationship between the NAO and August foraging was tested in two mixed effects models: one for winter and one for summer NAO, with individual as a random effect. A mixed effects model was also used to test whether any variation in mean January non-breeding latitude with changing ENSO conditions occurred as a result of individual adjustment. Between-individual and within-individual responses to ENSO conditions were separated using the subject centring method from van de Pol & Wright [24]. Finally, we implemented a mixed effects model to assess whether birds adjusted their non-breeding latitude to the latitude with the maximum chlorophyll in the Patagonian shelf from that year, with individual as a random effect. Significance was assessed in mixed effects models using likelihood ratio estimation and confidence intervals and effect sizes were obtained through bootstrapping methods; where 1000 simulations of random and fixed effects were implemented using the arm package [55].

## 3. Results

**Figure 2.**
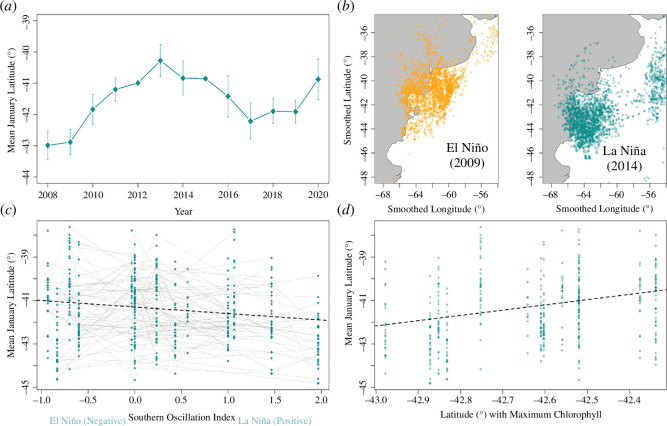
The effect of the ENSO index on non-breeding latitude. (*a*) Variation in mean non-breeding latitude (*n* = 422) between years with 95% confidence intervals, where each point represents a mean of all individuals. (*b*) Smoothed January latitude (°) and longitude (°) for all individuals are plotted for a strong El Niño year in orange (2009) (*n* = 36) and a strong La Niña year in blue (2014) (*n* = 37). (*c*) Variation in mean non-breeding latitude with the SOI index [28]. Grey lines connect individuals tracked over multiple years to visualize individual adaptation to varying ENSO conditions. The regression line is derived from the path analysis model. (*d*) The relationship between the latitude at which the maximum chlorophyll was centred for a given year against mean January latitude. Chlorophyll data were taken from the Aqua-MODIS project [47]. The regression line is derived from the mixed effects model.

The mean January latitude for non-breeding birds showed nonlinear variation between 2008 and 2020, oscillating in a wave-like pattern (figure 2). Consecutive years were more similar, with overlap between 95% CIs. This suggests that latitudinal shifts occur relative to the previous year, and birds are responding to a periodic environmental variable. Path analysis (figure 1) suggested this pattern may exist as a result of changes in ENSO; where a significant correlation existed between the SOI and non-breeding latitude (*β* = −0.331, s.e. = 0.084, *z* = −3.944, *p* < 0.001). Birds were observed further north during non-breeding in El Niño years (figure 2). To determine whether shifts occurred via individual flexibility in foraging latitude with ENSO, we implemented a mixed effects model via the subject centring method, to find both significant between (*β* = −0.77, 95% CI [−1.29,−0.28], *χ*^2^1 = 8.89, *p* < 0.01) and within individual effects (*β* = −0.30, 95% CI [−0.49,−0.12], *χ*^2^1 = 10.00, *p* < 0.01) [24]. There was, however, no significant difference in the within- and between-individual latitudinal change (95% CI [−1.01, 0.05], *p* > 0.05), suggesting that the effect of El Niño on non-breeding latitude is better explained by within-individual plasticity than between-individual turnover. We also implemented a separate mixed effects model that indicated birds adjust their non-breeding latitude to where the maximum chlorophyll that year was centred (*β* = 2.41, *χ*^2^1 = 47.24, 95% CI [1.71, 3.06], *p* < 0.0001). Therefore, birds appear to adjust their non-breeding latitude in response to shifting resource distributions.

**Figure 3.**
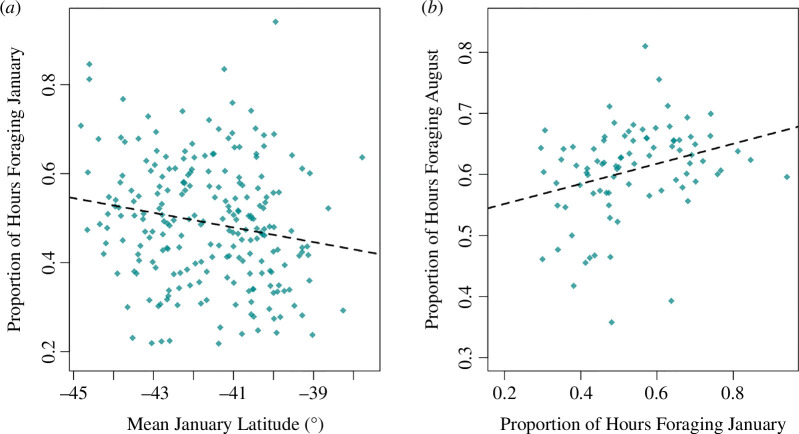
The correlative effects of January latitude on non-breeding and breeding foraging activity. (*a*) The proportion of the day spent foraging in January plotted against mean January latitude (*n* = 226). (*b*) The proportion of the day spent foraging in August during chick rearing plotted against the previous January’s foraging time (*n* = 87). For both, proportions are derived from foraging hours divided by the available daylight hours at the foraging site.

Variation in non-breeding latitude had a significant effect on the proportion of daylight hours spent foraging with foraging effort decreasing at lower latitudes (*β* = −0.0154, s.e. = 0.006, *z* = −2.517, *p* < 0.01). Therefore, ENSO-induced changes in January latitude appear to cause foraging efforts to vary. When January foraging effort was higher, it correlated with an increase in the proportion of time spent foraging in the following August (*β* = 0.216, s.e. = 0.080, *z* = 2.696, *p* < 0.01; figure 3). To summarize, northward shifts in foraging latitude as a result of El Niño conditions correlated with a reduction in non-breeding and subsequent foraging behaviour during breeding. Additionally, although based on a small sample size of non-breeders (*n* = 4), supplementary analysis indicated that reduced non-breeding (January) foraging effort may increase the propensity of birds to skip breeding (*β* = 12.87, s.e. = 5.67, *z* = 2.27, *p* < 0.05; electronic supplementary material 4). Foraging during August in year 0 had a positive relationship with the following January’s foraging (*β* = 0.940, s.e. = 0.146, *z* = 6.450, *p* < 0.001). We found no evidence of environmental conditions directly affecting foraging during breeding. We did not detect significant effects of ENSO on August foraging, nor winter (*β* = 0.06, 95% CI [−0.010, 0.018], *χ*^2^1 = 0.39, *p* > 0.05) or summer NAO (*β* = 0.01, 95% CI [−0.006, 0.03], *χ*^2^1 = 2.20, *p* > 0.05).

**Figure 4.**
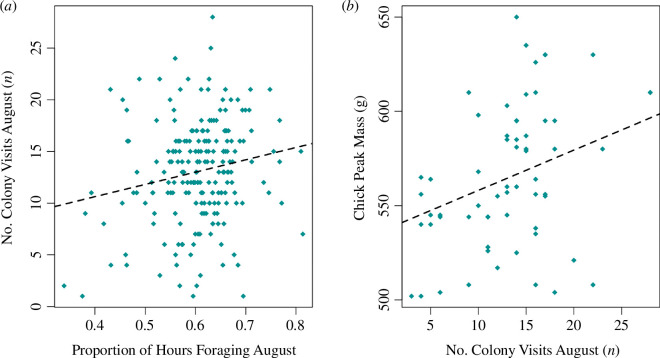
The correlative effects of foraging activity on colony visitation and chick peak mass. (*a*) The proportion of the day spent foraging in August (foraging hours divided by the available daylight hours at the foraging site) plotted against the number of colony visits in year 0 (*n* = 214). (*b*) The number of colony visits plotted against chick peak mass for Skomer birds only (*n* = 63). Regression lines are derived from (*a*) path analysis and (*b*) a mixed effects model.

An increase in foraging during August also increased the number of colony visits during this time (*β* = 12.681, s.e. = 4.831, *z* = 2.625, *p* < 0.01). We implemented a separate mixed effects model using chick peak mass measurements available from Skomer island (*n* = 63), to validate that the number of GLS-derived colony visits appear indicative of chick provisioning rates (*β* = 2.13, 95% CI [0.69, 3.60], *χ*^2^1 = 8.82, *p* < 0.01). There was no significant effect of the date peak mass was reached on the peak mass measurement (*β* = −9.52, 95% CI [−26.04, 8.38], *χ*^2^1 = 1.29, *p* = 0.2), indicating that varying breeding phonologies were not biasing August colony visitation rates. Therefore, increased colony visitation rates over August are correlated with higher chick peak body mass (figure 4). Neither foraging effort nor the number of colony visits in August had a significant effect on departure date from the colony (figure 1). Autumn southbound migration start date was, however, significantly related to the start date of the next spring’s northbound migration (*β* = 0.352, s.e. = 0.045, *z* = 7.808, *p* < 0.001), which defined return date to the colony in the following breeding season (*β* = 0.712, s.e. = 0.035, *z* = 20.491, *p* < 0.001). Therefore, individuals departing later on southbound migration from the colony appeared to return later next year. Neither the SOI nor non-breeding foraging effort had a significant effect on spring migration start date (figure 1). Migratory phenology, therefore, appeared separate to the effects of environmental variability via ENSO.

## 4. Discussion

Our main finding is that a long-distance migrant can adjust its non-breeding destination in response to large-scale oscillations in climate (ENSO). During El Niño years, birds did not travel as far south or spend as much time foraging during the non-breeding period as they did during La Niña. As we tracked individual birds across multiple years, we were able to demonstrate that this spatial response is primarily mediated by individuals flexibly adjusting their position with environmental conditions (and not the result of individual turn-over in our sample compounded by some sampling bias propagating through the annual cycle). Nevertheless, despite individual adjustments in position, reduced non-breeding foraging went on to impact the subsequent breeding attempt 10 000 km away, correlating with reduced foraging effort and colony visitation during chick-rearing (figure 2). These results suggest that El Niño conditions present losses in non-breeding foraging effort that may reduce physical condition during chick provisioning. Reduced non-breeding foraging effort may additionally increase the propensity of birds to skip breeding (electronic supplementary material figure S4), a result contrary to previous work that found the opposite effect in shearwaters [22] but examined data over a smaller number of years (and therefore ENSO conditions).

Reduced non-breeding foraging effort may plausibly occur via several mechanisms, for example, El Niño conditions may lower resource availability [56] or unfavourable environmental conditions may create reduced opportunities for foraging [57]. ENSO summarizes pressure differences that can equate to a range of environmental conditions including changes in sea level, ocean acidification, storms, sea surface temperature and precipitation; all known to impact seabird behaviour [4,58]. Such indices can allow ecologists to infer how seabirds might respond to climate without multiple hypothesis testing, with potentially more predictive power than local variables [59]. However, a northward shift in chlorophyll distribution has been documented along the Patagonian shelf during El Niño years and thought to be partially driven by wind anomalies [44]. We therefore conducted a secondary analysis (figure 2) using Aqua MODIS-derived chlorophyll a data that suggested birds are significantly shifting their latitude with where the maximum chlorophyll in the southwest Atlantic is centred [47]. Despite the limitations of using chlorophyll content as a proxy for prey distribution [60], this suggests that observed shifts in latitude are driven by prey availability [38]. Understanding how birds vary their behaviour with shifting resource distribution is important in terms of understanding current and future climatic changes, with future ENSO events predicted to become more extreme under greenhouse warming [61].

**Figure 5.**
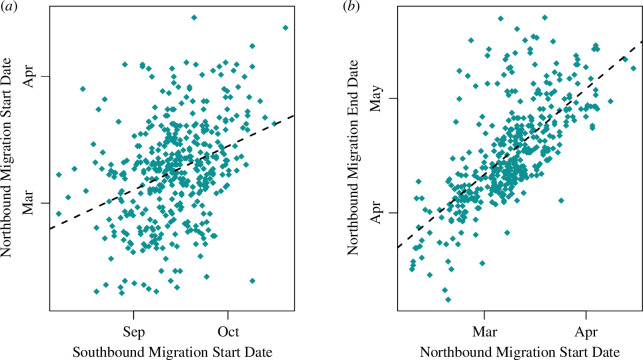
Correlations between southbound (autumn) and subsequent northbound migration (spring). (*a*) Northbound/spring migration start date plotted against previous southbound/autumn migration start date (*n* = 421). (*b*) Northbound/spring migration end date plotted against northbound/spring migration start date (*n* = 419). Regression lines are derived from the path analysis model.

Many avian species are seeing changes in breeding phenology so as to align breeding with peak prey availability [62]. However, we did not find any convincing effects of ENSO on migratory timings in Manx shearwaters (figure 2). Interestingly, migratory dates were highly correlated with one another (figure 5). We found that birds that leave earlier on southbound autumn migration do not have longer non-breeding periods but instead returned to the colony earlier next year. Breeding earlier is linked to higher success in many avian taxa, including shearwaters [63], so migratory timing may already be under strong directional selection [64–66].

Despite carry-over effects now being well documented in various taxa [15,67–69], including Manx shearwaters [10,22], the effects of global shifting environmental conditions are not well understood [70–72]. Here we tie carry-over effects to ENSO, yet do not find an influence of the NAO, a major driver of European climate, on breeding season behaviour. ENSO can influence northern hemisphere weather, and potentially even the NAO [73], so further research is needed to understand how environmental oscillations in different hemispheres may interact across the annual cycle of trans-global migrants.

## Data Availability

Data is available on Zenodo [74]. Supplementary material is available online [75].
